# Early fluid therapy with splanchnic sympathetic blockage prevented microcirculation damage, gut bacterial overgrowth, bacterial translocation and mortality in sepsis

**DOI:** 10.1186/cc11779

**Published:** 2012-11-14

**Authors:** AMA Liberatore, EC Del-Massa, LR Gondim, ALB Yamamura, IHJ Koh

**Affiliations:** 1 Federal University of São Paulo, Brazil

## Background

Herein we examined the hypothesis that preserving splanchnic microcirculation with lasting visceral parasympathetic vasodilatation plus a rapid hyperfluid therapy, during the early phase of severe sepsis (DL_60_), could delay and/or reduce the acutely exacerbating inflammatory feature of sepsis and modify its progression to death. In our previous work, this sepsis determined splanchnic tissue hypoxia, gut bacterial overgrowth, and bacterial translocation (BT) and these events were inflammatory amplification causal factors.

## Methods

Adult Wistar-EPM rats (*n *= 86) were anesthetized and submitted to: sepsis (S) (2 ml *Escherichia coli *10^8 ^CFU/ml i.v.) and/or thoracic epidural analgesia (E) (0.2 ml of 0.05% bupivacaine) and/or hyperfluid therapy (H) (30 ml/kg/20 minutes of Ringer lactate) according to the group (G) design: SG; SEG; SHG; SEHG; EG and; NG (naive). Epidural analgesia and fluid therapy were initiated 30 minutes after sepsis. At the 24-hour period, animals were monitored to: microcirculatory hemodynamics (duodenum, jejunum, ileum, liver and kidney) with sidestream darkfield imaging (SDF), gut Gram-negative bacterial count (duodenum, jejunum and ileum) and BT event in mesenteric lymph node (MLN), liver and spleen. Mortality was followed for 30 days.

## Results

**Figure 1:**
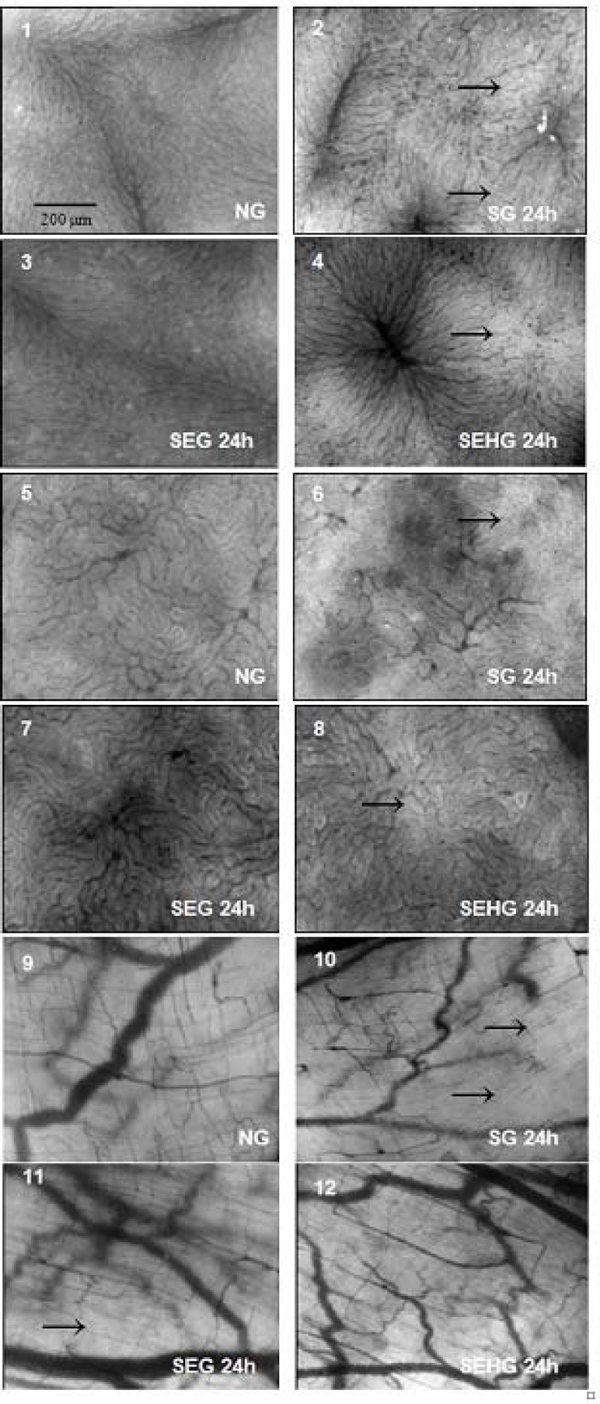
**SDF images of organs**. Liver (1 to 4) and ileum (9 to 12). Arrows indicate altered microvessels and perivascular tissue areas.

SDF findings showed that both EG and EHG preserved the microvascular and perivascular tissue architecture following sepsis induction in all organs. However, the SEH combination group showed larger microvessel diameter as compared with SEG, suggesting a better withholding of the intravascular blood volume subsequently improved tissue perfusion (Figure 1). A significant gut bacterial overgrowth was observed only in SG as compared with NG, EG, HG and EHG. The BT index to MLN was proportional to bacterial overgrowth, being higher in SG (67%), lower in SHG (33%) and SEG (17%), and absent in SEHG and EG. The mortality rate was 60% (SG), 30% (SEG) and 10% (HG). All animals with sepsis survived with E + H combination therapy.

## Conclusion

The splanchnic sympathetic blockage by epidural analgesia attenuated abdominal organs' microcirculatory dysfunction by augmentation of the parasympathetic vasodilatation effect, which might have preserved gut microcirculation, minimizing bacterial overgrowth, BT and subsequent gut immune activation. In addition, vigorous fluid therapy in the early phase of sepsis might have modulated the host systemic inflammatory response by ensuing better tissue perfusion. The combined therapy, by acting differently via an inflammation trigger, might have promoted a synergistic effect. Studies are in progress to verify the combined therapy potentials in the later phase of sepsis.

